# Supplementary material for: Transcriptomic Profiling of Rectus Abdominis Muscle in Women with Gestational Diabetes-Induced Myopathy: Characterization of Pathophysiology and Potential Muscle Biomarkers of Pregnancy-Specific Urinary Incontinence
Source: Int J Mol Sci. 2022 Oct 25;23(21):12864. doi: 10.3390/ijms232112864 (PMC9658972; doi:10.3390/ijms232112864)
Supplement: Supplementary file 1 [file ijms-23-12864-s001.zip › Supplementary Materials- legends.pdf]

Table S1. Normalized counts of all genes identified in the 12 samples divided into four groups

Table S2. List of differentially expressed genes in NG-C, NG-I and GDM-C women when compared with GDM-I women. The genes were considered differentially expressed when log2 Fold Change ≥ |1.5| and the adjusted p-value (padj) ≤ 0.05. Purple color indicates genes with down-regulated transcription and orange color indicates genes with up-regulated transcription in GDM-I. The genes are identified by the symbols and full names.*ND-C: non-GDM women and continent women- no PSUI; ND-I: Non-GDM women and incontinente women- with PSUI; GDM-C: GDM women and continent women- no PSUI; GDM-I: GDM women and incontinent women- with PSUI.

Table S3. List of genes included in Venn diagram. The list of differentially expressed genes in Venn diagram with transcription when compared the three control groups (ND-C, ND-I and GDM-C) with GDM-I. The genes were up-regulated in GDM-I (orange) or down-regulated in GDM-I (purple). The genes are identified by the symbols (the full names can be found in the table S2).The genes are identified by the symbols (the full names can be found in the table S1).*ND-C: non-GDM women and continent women- no PSUI; ND-I: Non-GDM women and incontinente women- with PSUI; GDM-C: GDM women and continent women- no PSUI; GDM-I: GDM women and incontinent women- with PSUI.

Figure S1. Transcriptome principal component analysis (PCA) of RAM samples of four groups analyzed in this study. PCA plot showing gene expression data grouped according to the ND-C, ND-I, GDM-C and GDM-I. Principal components were obtained using the log2 normalized counts for the genes in each sample.

Figure S2. Reproducibility of reference controls: The constancy of the expression level of ACTB and GAPDH in the different groups (shown by similar Ct values).
