# Supplementary figures and images for: Transcriptomic Profiling of Rectus Abdominis Muscle in Women with Gestational Diabetes-Induced Myopathy: Characterization of Pathophysiology and Potential Muscle Biomarkers of Pregnancy-Specific Urinary Incontinence
Source: Int J Mol Sci. 2022 Oct 25;23(21):12864. doi: 10.3390/ijms232112864 (PMC9658972; doi:10.3390/ijms232112864)

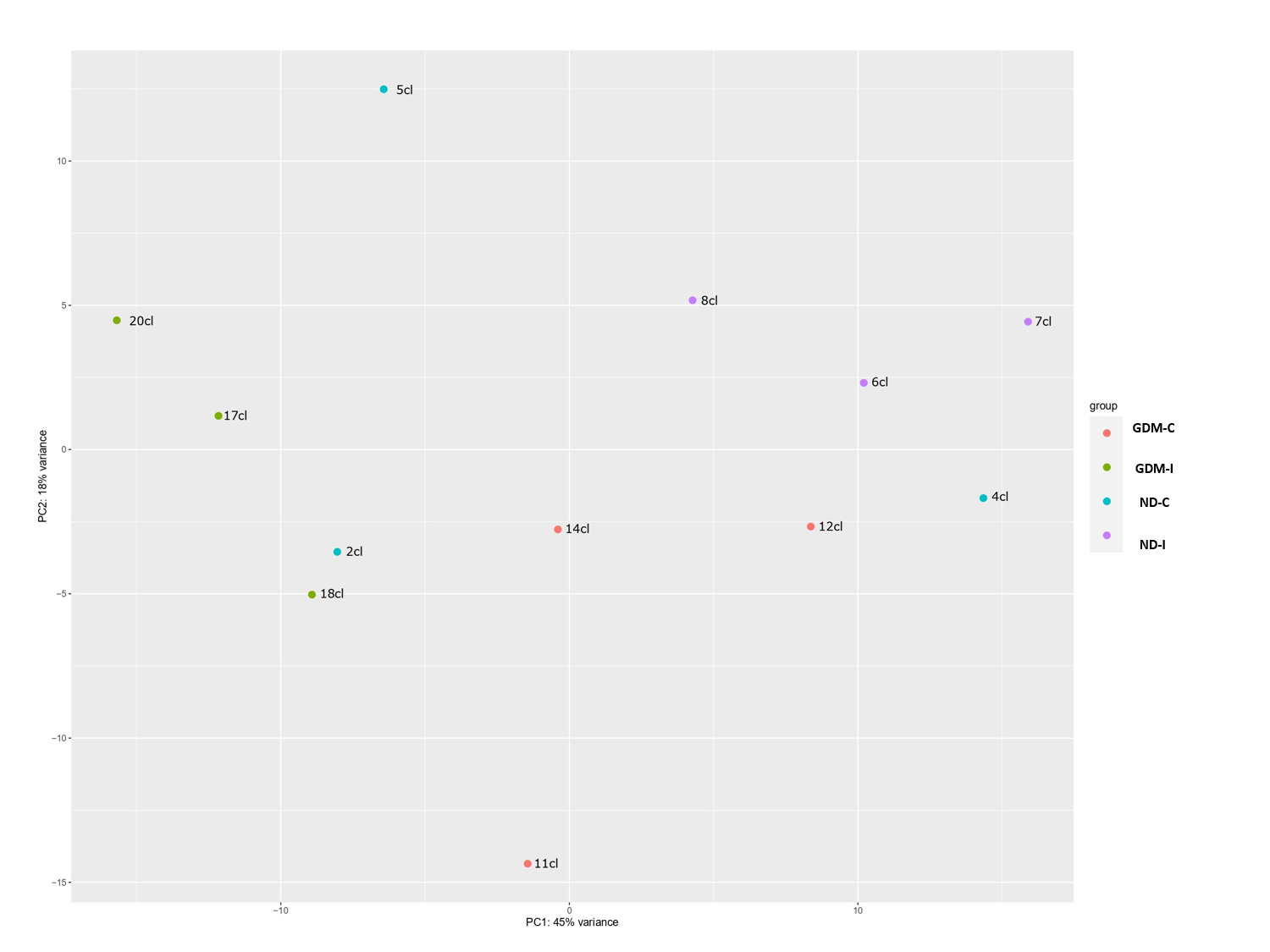

Supplement: Supplementary file 1 [file ijms-23-12864-s001.zip › Figure S1- PCA.png]

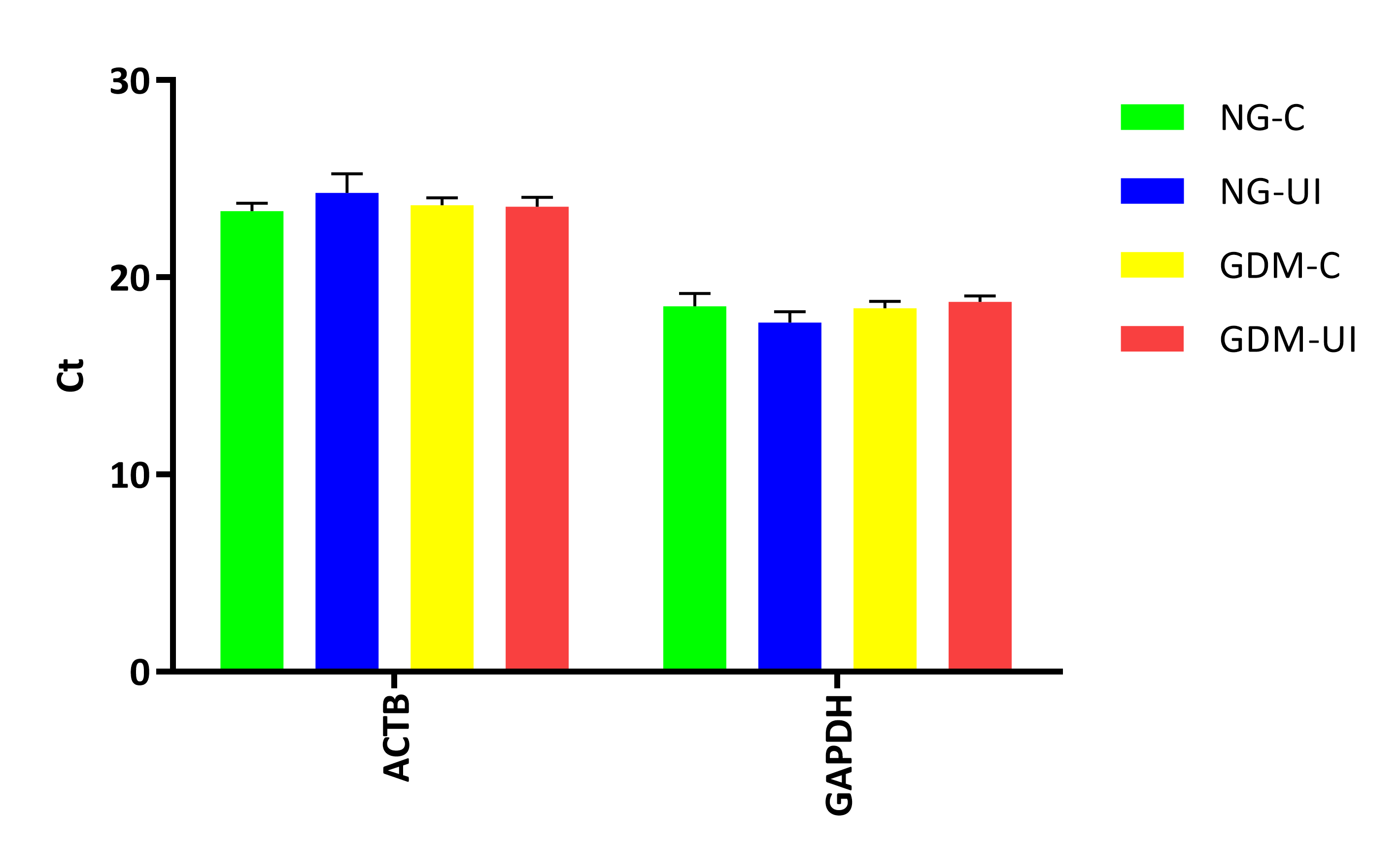

Supplement: Supplementary file 1 [file ijms-23-12864-s001.zip › Figure S2.png]
